# Prone ventilation of critically ill adults with COVID-19: how to perform CPR in cardiac arrest?

**DOI:** 10.1186/s13054-020-02970-y

**Published:** 2020-05-26

**Authors:** Wioletta Mędrzycka-Dąbrowska, Katarzyna Lewandowska, Daniel Ślęzak, Sebastian Dąbrowski

**Affiliations:** 1 Department of Anaesthesiology Nursing and Intensive Care, Medical University of Gdansk, Dębinki 7, 80-211 Gdansk, Poland; 2 Department of Emergency Medicine, Medical University of Gdansk, Gdansk, Poland; 3 Department of Anesthesiology and Intensive Care, Specialistic Hospital, Gdańsk-Zaspa, Poland

Dear Editor,

We read with great interest an editorial by Siow et al. on managing COVID-19 in resource-limited critical care settings [1]. As critically ill COVID-19 patients seem to benefit from prone position ventilation, we think it would be worth mentioning another clinical intervention that—performed in this position—would limit workload and reduce exposure risks of the staff, i.e., CPR. The idea of “prone-CPR” is not novel. It was first introduced by McNeil in 1989 and followed by some other authors publishing research papers and case reports [2, 3], but it did not gain significant traction among medical professionals. There are some instances though when there may be little alternative to CPR in the prone position, e.g., cardiac arrest in neurosurgical patients, when the brain or spinal cord are exposed during surgery and turning to the conventional supine position would cause neural damage [3, 4]. We believe that cardiac arrest in a prone-ventilated patient with COVID-19 may be another indication for commencing CPR without de-proning. There seem to be some undisputable advantages of performing prone cardiopulmonary resuscitation in this particular group of patients. Firstly, it would limit the amount of people being exposed to environment with highly contagious material. Some guidelines suggest that optimal staffing involved in turning a critically ill prone patient should count minimum of five people and may take up to 5 min to be completed, and time during cardiac arrest is of the essence. Secondly, there is a risk of displacement of the endotracheal tube or even inadvertent extubation of the trachea which might have disastrous consequences for the patient and the staff (aerosol-generating incident). Disconnection of vascular lines as well as injury to the patient and staff might occur.

During prone-CPR, chest compressions may be performed placing hands over each scapula or over the thoracic spine with or without counter-pressure on the sternum. Successful defibrillation has also been described with several pad positions [4]..

In the current epidemiological situation, understaffed ICU teams should be prepared for the situations which are far from the daily routine. While the effectiveness of CPR in the prone position is not completely known, we think that the prone-CPR should be given a consideration during a sudden cardiac arrest in COVID-19 victims being prone-ventilated. This idea was also reflected in the recently published online interim guidance for BLS and ALS in patients with suspected or confirmed COVID-19 [5].

## Data Availability

Not applicable.
